# 

**Published:** 1898-11

**Authors:** 


### CONTENTS.

COMMUNICATIONS.

The Importance of Establishing a Technic as well as Literary Standard for College Entrance .......... 501
Method of Teaching Materia Medica; Abstract .......... 507

PROCEEDINGS.

National Dental Association, Omaha, Nebraska, 1898 .......... 510
Cast and Model .......... 511
Materia Medica and Therapeutics .......... 512
Anatomy, Pathology and Surgery .......... 514
The Comparative Method of Teaching Dental Anatomy .......... 522
Resolutions Adopted by the National Dental Association at the Annual Session of 1898, and Other Miscellaneous Items .......... 530

SELECTIONS.

After a Fracture of the Limb the Nails of the Hand on the same side Cease Growing .......... 506
Orthoform .......... 536
How to Conquer Pain .......... 537
Enforced Higher Education .......... 538
Orthoform, A New Local Anesthetic .......... 539
Insomnia and Suffering .......... 539
Cure for Headache .......... 540
A Whiff of Tobacco and Its Unpleasant Suggestion .......... 541
The Psychology of Laughing .......... 542
Hygiene of the Teeth .......... 543
Disinfection of Hands .......... 543
To Cure an Ingrowing Toe-nail .......... 544
Finishing Proximal Cement Fillings .......... 544
Toothache .......... 545
Alcohol Consumption in the United States on the Decrease .......... 545

NOTICES.

To the Members of the Ohio State Dental Society .......... 545

EDITORIAL.

Bibliographical .......... 546
Information .......... 548
Suspension—American Dental Weekly .......... 549
A New College .......... 550
The Board of Dental Examiners .......... 550

